# Distinct Clinical and Laboratory Features of Measles in Adults and Children During the 2024 Epidemic: A Retrospective Study from a Romanian Tertiary Infectious Diseases Center

**DOI:** 10.3390/medicina62050836

**Published:** 2026-04-28

**Authors:** Andrei Vâţă, Ionela-Larisa Miftode, Maria Gabriela Grigoriu, Ioana Mihuta, Ioana Maria Onofrei, Alexandru Florinel Oancea, Mihaela Catalina Luca, Egidia Gabriela Miftode

**Affiliations:** 1 Faculty of Medicine, “Grigore T. Popa” University of Medicine and Pharmacy, 700115 Iasi, Romania; andrei.vata@umfiasi.ro (A.V.); grigoriumaria.mg@gmail.com (M.G.G.); maria-ioana.hunea@umfiasi.ro (I.M.O.); alexandru.oancea@umfiasi.ro (A.F.O.); mihaela.luca@umfiasi.ro (M.C.L.); egidia.miftode@umfiasi.ro (E.G.M.); 2 “St. Parascheva” Clinical Hospital of Infectious Diseases, 700116 Iasi, Romania; ioanaminodoram@gmail.com

**Keywords:** measles outbreak, Romania, hepatic involvement, pneumonia, vaccination coverage, adults, children, epidemiology, complications

## Abstract

*Background and Objectives*: Romania reported the highest measles incidence in the European Union during the 2023–2024 epidemic, largely driven by declining vaccination coverage. We aimed to characterize the epidemiological, clinical, and laboratory profile of hospitalized measles patients and to identify age-related differences, with particular emphasis on systemic and hepatic involvement. *Materials and Methods*: We conducted a retrospective observational study including 360 consecutive patients with laboratory-confirmed measles admitted to a tertiary infectious disease hospital in northeastern Romania between 1 January and 31 December 2024. Demographic, clinical, laboratory, therapeutic, and outcome data were collected. Pediatric (<15 years) and adult patients were compared using appropriate statistical tests. *Results*: Children accounted for 71.4% of cases, including 16.1% infants under one year. Over 90% of patients were unvaccinated or incompletely vaccinated. Household transmission represented the most frequent identifiable source. Adults presented significantly higher inflammatory markers and more pronounced hepatic involvement than children. ALT elevation occurred in 63.1% of adults versus 34.2% of children (*p* < 0.001), with moderate-to-severe cytolysis predominantly observed in adults (34.9% vs. 1.9%, *p* < 0.001). Pulmonary complications were documented in 28% of cases, mainly viral interstitial pneumonia. Thrombocytopenia was significantly more frequent in adults (*p* < 0.001). Overall mortality was 0.27%, occurring in an unvaccinated infant with secondary bacterial pneumonia. *Conclusions*: The 2024 measles epidemic in our area was characterized by sustained transmission among unvaccinated individuals and frequent systemic involvement. Hepatic dysfunction emerged as a prominent feature in adults, suggesting a shifting clinical phenotype in contemporary outbreaks. Strengthening vaccination coverage and early recognition of systemic complications remain critical to reducing measles-related morbidity and mortality.

## 1. Introduction

Measles is an acute viral infectious disease, universally spread and highly contagious, which has recently become a major public health problem globally, especially in populations with suboptimal vaccination coverage [1,2]. The measles epidemic that started in 2023 represents the largest re-emergence of the disease recorded in Romania in the last decade and is part of a European context marked by the resurgence of sustained transmission of the measles virus [3,4]. According to data published by the World Health Organization—European Region and the European Centre for Disease Prevention and Control—2024 saw an increase of over 300% in the number of measles cases in the European region compared to the previous year, Romania becoming the main regional source of infection [3,5]. Comparative analyses across Europe indicate that regions with vaccination coverage below 90% face a risk of outbreak occurrence more than five times higher than regions reaching the 95% threshold [6,7]; in Romania, at the beginning of 2025 the vaccine coverage with two doses of MMR at age of 5 was estimated at 55.1% [8].

Measles infection manifests with a characteristic prodrome of fever, cough, coryza, and conjunctivitis followed by a maculopapular rash in both children and adults; however, the clinical severity and constellation of complications diverge between age groups. In adults, especially during the recent Romanian epidemic, high frequencies of systemic inflammatory responses and end-organ involvement have been reported [9]. A Romanian single-center cohort demonstrated that hepatic cytolysis (elevated AST/ALT) was present in a large proportion of hospitalized adults and correlated with more severe disease progression, reflecting a distinctive feature of adult measles not typically emphasized in pediatric cohorts [10]. Adults also exhibited near-universal elevated C-reactive protein levels and lymphopenia upon admission, which are indicators of systemic inflammation and immune suppression that may predispose to secondary bacterial complications and respiratory failure [10].

By contrast, pediatric measles cases in Romania were characterized by a high burden of respiratory and gastrointestinal complications, with pneumonia and enterocolitis occurring more frequently compared to adults in earlier epidemic waves, and with increased rates of severe disease among very young children lacking maternal antibody protection or vaccination [11]. Epidemiological data from pediatric cohorts documented shifts toward older children over successive epidemic waves, underscoring immunity gaps in the vaccinated pediatric population and reinforcing that disease patterns may evolve as vaccination coverage fluctuates [12]. Laboratory investigations in pediatric cases often reveal leukopenia, thrombocytopenia, and elevated inflammatory markers, but the emphasis in younger age groups has traditionally been on respiratory and otolaryngologic complications rather than hepatic involvement [11,12].

Complication profiles also reflect age-related pathogenetic mechanisms. Pneumonia remains the most common serious complication across all ages, yet adults appear particularly prone to acute respiratory failure requiring supportive care, especially in the presence of chronic comorbidities [10,12]. Meanwhile, severe pediatric complications include otitis media, dehydration, and malnutrition-related morbidity [13,14]. Long-term sequelae such as subacute sclerosing panencephalitis, although rare, are predominantly sequelae of measles acquired in early childhood rather than adult infection, reflecting age-dependent neuropathological vulnerability [13].

Despite therapeutic advances, measles continues to impose a considerable burden on health systems, with more than half of reported cases requiring hospitalization, particularly among children [3,15].

This study aimed to characterize the epidemiological, clinical, and laboratory profile of hospitalized measles patients and to identify age-related differences, with particular emphasis on systemic and hepatic involvement.

## 2. Materials and Methods

This study was designed as a retrospective, single-center observational study conducted at “St. Parascheva” Clinical Hospital of Infectious Diseases, Iași, Romania, a tertiary referral center for infectious diseases.

### 2.1. Study Population

We included 360 consecutive laboratory confirmed measles cases from our hospital, between 1 January and 31 December 2024. Measles diagnosis was established based on compatible clinical criteria (fever, maculopapular rash, and associated symptoms) and laboratory confirmation, in accordance with national surveillance definitions—detection of measles-specific IgM antibodies (Biosan^®^ ELISA line, Biosan, Riga, Latvia) and/or measles virus RNA by molecular testing (CFX96^®^ System, Bio-Rad Laboratories, Hercules, CA, USA).

Patients of all ages were eligible for inclusion. Children were defined as individuals under 15 years of age, in accordance with World Health Organization measles surveillance standards. For ethical purposes, pediatric participants were considered as persons under 18 years of age.

Re-admitted patients or without hematological and biochemical laboratory data were excluded (6 cases).

### 2.2. Data Collection

Data were retrospectively extracted from electronic and paper medical records using a standardized data collection form.

The following variables were recorded: demographic data—age, sex, and area of residence (urban or rural); clinical characteristics—fever, cough, rhinorrhea, conjunctivitis, Koplik spots, gastrointestinal symptoms, day of disease at hospital admission, and day of rash at admission; laboratory parameters at admission—complete blood count (leukocyte count, neutrophil percentage, platelet count), C-reactive protein (CRP), aspartate aminotransferase (AST), alanine aminotransferase (ALT), and total bilirubin; therapeutic interventions—antibiotic therapy and oxygen supplementation; clinical outcomes—development of complications and lethality.

The vaccination history was assessed using the National Electronic Vaccination Registry and for those born before 2011, by consulting the individual vaccination cards.

The clinical cases were classified as: mild measles—typical presentation, no organ involvement; moderately severe measles—more intense symptoms with mild complications; severe measles—major complications or organ involvement (pneumonia (viral or secondary bacterial), acute encephalitis, severe thrombocytopenia).

Hepatic involvement was defined based on serum ALT levels. An elevation above the upper limit of normal (ULN; 34 UI/L, regardless of the patient’s age) was considered indicative of liver involvement and classified as mild (ALT ≤ 5× ULN), moderate (ALT > 5× to ≤10× ULN), or severe (ALT > 10× ULN). ALT values were assessed at admission and during hospitalization when available, with or without clinical signs of hepatitis. Peak levels during hospitalization were used in the analysis.

Measles-associated pneumonia was defined as the presence of clinical signs of lower respiratory tract infection occurring during acute measles, supported by radiological findings (DMS Platinum Neo dRF system) and/or hypoxemia. Cases were classified as viral or bacterial pneumonia based on timing and radiological data (interstitial infiltrates vs. lobar opacity or bronchopneumonia).

### 2.3. Statistical Analysis

Statistical analyses were performed using SPSS 31.0 software. Continuous variables were tested for normality and are presented as mean values. Comparisons between patients were performed using the independent samples *t*-test for normally distributed variables and the Mann–Whitney U test for variables with non-normal distributions. Categorical variables were analyzed using Fisher’s exact test. A two-sided *p* value of <0.05 was considered statistically significant.

### 2.4. Ethical Considerations

The study was conducted in accordance with the Declaration of Helsinki and approved by the local ethics committee of “St. Parascheva” Clinical Hospital of Infectious Diseases, Iași, Romania. At hospital admission, the patients or their parents (for those less than 18 years old) signed an informed consent allowing us to use their anonymized medical data for scientific purposes.

## 3. Results

**Figure 1:**
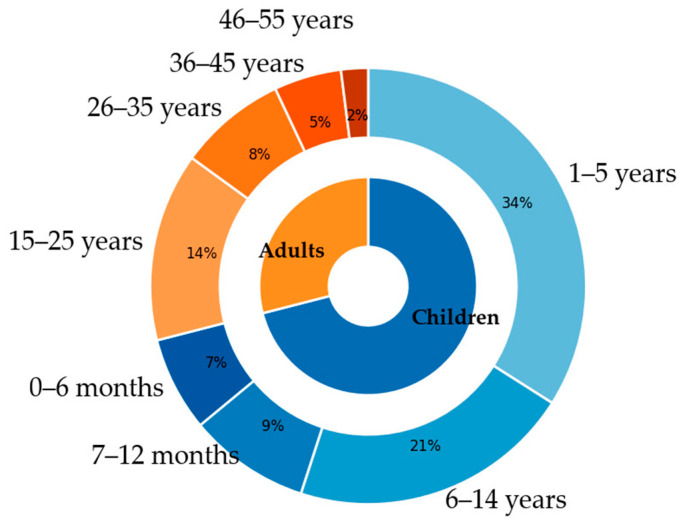
Age group distribution of the included patients.

A total of 360 patients hospitalized with laboratory-confirmed measles (257 children and 103 adults) (Figure 1) were included in the analysis. The median age was 11 years (interquartile range: 2–32 years), with a slight predominance of male patients. 71.4% of the patients were children with a median age of 3 (interquartile range: 1–6 years), 22.6% under the age of one year; the children M/F ratio was 1.03. The four youngest patients were only 2-month-olds. The median age of the included adults was 26 years (interquartile range: 19–36 years), with a maximum of 51 and the M/F ratio 0.71.

**Figure 2:**
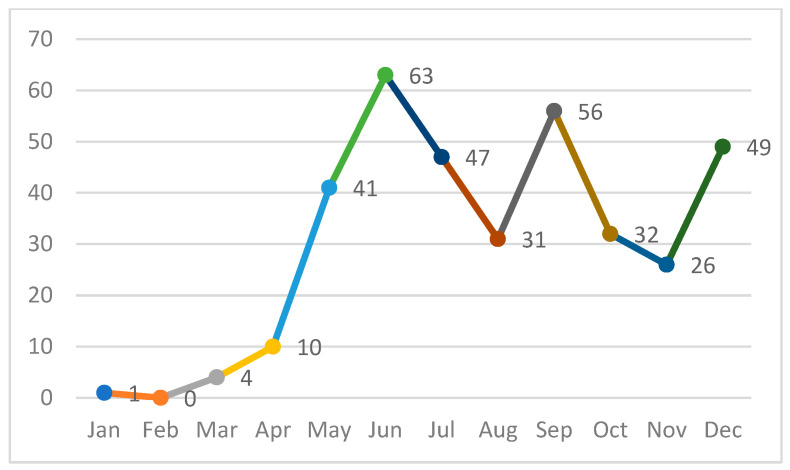
Monthly distribution of measles cases.

Although Romanian health authorities declared a measles epidemic on 5 December 2023, the widespread virus transmission continued throughout 2024; in our hospital, the case numbers began to significantly rise in May and consistent numbers persisted until the end of the year (Figure 2).

Data regarding measles vaccination could be confirmed in 88.9% of the patients. 59 of them had a history of previous immunization. 23 of the latter had only incomplete vaccination (one dose). The mean age of the patients who previously received a complete course of measles vaccination (two doses) was significantly higher than the rest of the lot (17 vs. 11 years, *p* < 0.001).

The source of the infection could be identified in 41.7% of cases; most frequent it was a member of the same family (77.3% of known cases). 16.1% of the hospitalized cases were too young to have begun their immunization schedule.

### 3.1. Clinical Picture

Most patients were admitted median 4 days after disease onset and 2 days after rash onset, indicating a variable delay in presentation to medical care. No differences were identified between children and adults.

The main clinical manifestations and their frequency are presented in Table 1.

36.9% of the children and 49.5% of the adults experienced during their illness a body temperature above 39 °C (*p* = 0.028).

The laboratory data at admission are presented in Table 2. The children had an inflammatory response with more leukocytes and less neutrophils than the adults. The CRP levels were higher in adults.

### 3.2. Measles-Related Complications and Treatment

According to disease severity, 12.8% of cases were mild, 83.9 were moderately severe and 3.3% were severe. The proportion of severe cases was similar between children and adults (11.9 vs. 10.3, *p* = 0.77).

One death was reported (lethality 0.27%)—an unvaccinated 8-month-old male child, with no previous comorbidities, who developed a secondary bacterial pneumonia and despite intensive care and antibiotics died after a prolonged hospitalization.

Almost half (44.4%) of the patients had ALT elevations at admission or during their hospitalization (Table 3). The hepatic involvement was more common in adults, as frequency (63.1 vs. 34.2, *p* < 0.001) and intensity (34.9 vs. 1.9% moderate and severe ALT elevation, *p* < 0.001).

Pulmonary complications were identified in 28% of cases. Interstitial (viral) pneumonia was the most common finding (74.2% of all pneumonia cases, followed by lobar pneumonia 17.8% and bronchopneumonia 7.9%). 3.3% of patients developed respiratory insufficiency (eight children and four adults) and required non-invasive supplemental oxygen therapy. One required mechanical ventilation.

Thrombocytopenia was present in 18.3% of all cases The proportion of cases was significantly higher in adults compared to children (34/103 vs. 32/257; χ^2^ = 12.1, *p* < 0.001).

Other reported complications were: neurological—acute encephalitis (two cases), generalized seizures (one case), secondary acute sinusitis (six cases), acute otitis (five cases).

Besides symptomatic treatment, most patients (93%) also received antibiotics during their hospitalization. No significant differences between the frequency of antibiotic administration were detected between children and adults (94.5 vs. 89.3%, *p* = 0.07). The most common antibiotics administered were Ampicillin—45.9% of cases and Ceftriaxone—37.3% of cases. The mean duration of the antibiotic treatment was 5 days.

## 4. Discussion

The measles epidemic in Romania, which began in late 2023, reached an exceptional magnitude, representing the largest resurgence of the disease at both national and European levels in recent decades. Following a period of reduced incidence during the COVID-19 pandemic, measles transmission increased progressively in the second half of 2023 and early 2024, in parallel with broader trends observed across the European Union and European Economic Area [6]. Romania became the epicenter of the regional epidemic, reporting 30,692 cases in 2024 and accounting for approximately 87% of all notified cases in the EU/EEA, with a notification rate of 1610.7 cases per million population [6]. Between February 2024 and January 2025, more than 27,500 cases and at least 18 measles-related deaths were reported nationally, underscoring the substantial public health burden of the outbreak [6]. Transmission remained sustained throughout 2025, with more than 8000 additional cases reported by autumn, although with a declining trend compared to the previous year [6]. This epidemic was characterized by widespread endemic transmission, disproportionate involvement of young children, high hospitalization rates, and measurable mortality, reflecting persistent gaps in vaccination coverage and reaffirming Romania’s role as the primary measles hotspot in Europe in the post-pandemic period [15].

This single-center observational study provides a comprehensive clinical and biological characterization of hospitalized measles patients during the current epidemic wave in Romania, with a particular focus on differences between children and adults.

Compared with the 2011–2012 epidemic and the 2016–2020 epidemic wave, the 2024 outbreak was characterized by intensified community transmission and a marked shift in age distribution, with a higher burden of disease among infants, adolescents, and young adults [6].

According to the ECDC, in 2024, measles impacted all demographics, though infants under the age of one faced the highest risk with a notification rate of 1175.4 per million. Young children aged 1–4 followed closely, with a rate of 688.7 per million. The data also reveals a significant shift toward older populations—individuals over 14 accounted for 26% of all cases. In several countries, adults over 30 made up the majority of reports, ranging from 28% to 53% [6]. In our study, most patients were children <14 years—71.4%. The most affected age group was young children aged 1–4—34.7% of cases, followed by children aged 6–14—20.6%. Infants represented 16.1% of the patients. In adults, those aged 15–25 were the best represented group with 13.9%. A similar pattern was described in a pediatric Romanian population by Jugulete et al. [11]. They also showed that age group distribution can vary between epidemic waves.

More than 85–90% of reported cases in the 2024 epidemic in Romania occurred in unvaccinated individuals or in those with incomplete vaccination status, underscoring the decisive role of insufficient vaccination coverage in sustaining measles virus transmission [6,16]. A recent comparative analysis of the 2017–2019 and 2023–2024 epidemic waves have documented a significant rise in measles incidence among unvaccinated children, accompanied by a progressive shift in cases toward older age groups, a pattern attributed to persistent immunization gaps [11]. In our cohort, documenting the vaccination history was difficult, data being obtained in 88.9% of cases. Measles appeared mostly in unvaccinated individuals—90% of all cases. The same percentage is mentioned in the 2025 ECDC report [6].

Lazar et al. [17] who analyzed the measles outbreaks in Romania (March–August 2023) conclude that the major source of infection was not an isolated external one, but an endemic transmission caused by insufficient vaccination coverage, which led to the emergence and maintenance of transmission chains between susceptible individuals. In this analysis, 78% were unvaccinated, a significant number were too young to be vaccinated, and some had an incomplete vaccination schedule.

The occurrence of measles in vaccinated individuals is rare, but possible. The main explanations are primary vaccine failure, waning immunity or intense exposure during outbreaks [18,19]. The higher mean age of the patients who previously received a complete course of measle vaccination among our lot could suggest waning immunity over time.

From an epidemiological point of view, measles is seasonal, with increased incidence in the winter and spring months, a phenomenon consistently observed in European studies and correlated with population density and intense circulation of respiratory viruses [15,20]. In populations with insufficient vaccination coverage, this seasonal pattern favors the emergence of widespread outbreaks, with sustained transmission over several months. In our study, the number of cases began to rise in May, with peaks in June (17.5% of cases), September (15.5%) and December (13.6%), lacking the usual seasonality.

From a clinical perspective, the recent epidemic in Romania was characterized by increased severity compared to previous waves, reflected in high hospitalization rates and the frequency of complications [16,21]. Data reported by infectious disease hospitals indicate hospitalization rates exceeding 50–55% in certain counties, with higher values in infants and patients with comorbidities. Respiratory complications were the most common, reported in over 75–80% of hospitalized children and over 60% of adults, including severe viral pneumonia and bacterial superinfections [16]. Compared to the 2016–2020 epidemic, there was an increase in the proportion of severe cases requiring intensive monitoring [16,21].

In adults, measles often follows a more severe course than in children, with a higher rate of complications and hospitalizations [1,2,13,22,23].

Measles in adults is frequently underdiagnosed in its early stages because the prodromal symptoms are similar to those of other respiratory viral infections. This contributes to delays in isolating cases and increases the risk of nosocomial transmission, a phenomenon documented in multiple hospital outbreaks reported in the European literature [13,15]. Our analysis of the time to hospitalization indicates a similar presentation pattern across age groups. In both the pediatric and adult populations, hospitalization occurred at a median of 4 days from the onset of symptoms, with no differences between the two groups. This consistency suggests that clinical manifestations (such as rash) triggered the decision to see a doctor equally for children and adults.

The initial clinical manifestations in adults are similar to those observed in children, including high fever, cough, rhinorrhea, and conjunctivitis, but the duration of the prodromal phase is often longer and the intensity of symptoms more pronounced [13]. Clinical studies report fever values exceeding 39–40 °C in more than 65% of adult patients, compared to approximately 45–50% in children [1,22]. In our study, more than 93% of the patients reported fever during their illness, and its intensity was significantly higher in adults (*p* = 0.01) (Table 1). Also, a higher proportion of the adults presented fever higher than 39 °C than the children (*p* = 0.028). Being a retrospective study, we had no quantitative data to allow us to compare the general toxic syndrome or the intensity of the rash. Gastrointestinal involvement is also common in adults, with acute diarrhea reported in 30–60% of patients, a lower percentage than in children, but with a more severe clinical impact due to the increased risk of dehydration and electrolyte imbalances [1,15]. Cases of severe enterocolitis and acute pancreatitis associated with measles have been described, particularly in immunocompromised adults, although their incidence remains below 5% [13,23]. In our cohort, rhinorrhea was significantly more common in children (*p* < 0.001), as was the presence of diarrhea (*p* = 0.054), clinically significant adenopathies (*p* = 0.001) and conjunctivitis (*p* < 0.001).

A central element in the pathogenesis of measles is the ability of the virus to induce profound and transient immunosuppression [1,24]. This impairment explains the increased susceptibility to secondary infections (both bacterial and viral), which can occur both in the acute phase and in the weeks or months following clinical recovery. In children, the immaturity of the immune system amplifies these pathogenic mechanisms. Clinical studies show that the cellular immune response is less effective in young children, which favors extensive viral replication and systemic dissemination [2,13].

Hematological impairment is common in children and adults with measles. Leukopenia, anemia, and thrombocytopenia have been reported in 38–47% of pediatric cohorts analyzed during recent epidemics [3,24]. Observational studies have reported leukopenia in approximately 40–60% of patients and thrombocytopenia in 10–25%, with these changes being correlated with disease severity and the risk of bacterial superinfections [1,24]. I our cohort, the average number of leukocytes at admission was significantly lower in adults, as was their thrombocytes number (*p* < 0.001) (Table 2). The percentage of the neutrophils was higher in adults. Several recent immunology reviews discuss the role of components of the innate system (including neutrophils) in response to the measle virus, although the clinical details related to the precise number of neutrophils in the disease remain relatively poorly studied compared to other viral infections [25].

Liver damage in measles is the result of the interaction between systemic viral replication and the host’s immune response, being determined both by the direct cytopathic effect of the measles virus on hepatocytes and by secondary immunopathological mechanisms. Histopathological evidence supports the direct involvement of the virus at the hepatic level, with areas of focal or diffuse hepatocyte necrosis described, without marked portal inflammation, as well as the detection of viral RNA in hepatocytes by molecular techniques, suggesting a direct cytotoxic effect [26]. In addition to this direct mechanism, liver damage is amplified by the intense systemic immune response characteristic of measles infection. In the cohort analyzed in the 2022–2024 epidemic in Romania, severe lymphopenia was identified in 51% of patients with hepatocytolysis, compared to 35.7% of patients without liver damage [27].

Liver damage is common in adults with measles but is much rarer and milder in children: elevated transaminases are reported in 50–80% of adult patients, with most cases being transient, but severe forms of measles hepatitis are described particularly in patients with pre-existing liver disease [1,13]. Our study showed that 44.4% of the patients had ALT elevations during their illness and that the adults had this problem with a higher frequency (63.1% vs. 34.2%, *p* < 0.001) and intensity (mean ALT 155 vs. 44 UI/L, *p* < 0.001). Clinical studies indicate that alanine aminotransferase levels may exceed 3–5 times the upper limit of normal in approximately 20% of patients, without being associated with liver failure in most cases [2,22]. Moderate and severe ALT elevation were more common in adults in our study (34.9 vs. 1.9%, *p* < 0.001).

A more than usual hepatic involvement in recent outbreaks from Romania was also reported by Niculae et al. [27]. These findings suggest a shift in the clinical profile of measles in the context of circulation of the current epidemic strains. Furthermore, significant hepatocellular injury, defined as ALT values ≥ 10 times the upper limit of normal, was reported in 23.6% of patients during the 2022–2024 epidemic, representing nearly double the proportion observed in the previous outbreak (10.7%; *p* = 0.003). This rate is considerably higher than that reported in earlier studies, in which severe hepatic involvement was described in fewer than 6% of patients, underscoring the unusually severe nature of liver involvement in the current epidemic. Concordant findings were also reported in another recent observational study conducted in Romania, in which elevated AST levels were observed in 87.3% of adult patients with measles, while ALT elevations were documented in 76% of cases [9]. Clinical jaundice was present in only 12.7% of patients, confirming the predominantly subclinical nature of hepatic involvement. In the same study, cholestatic abnormalities, reflected by increased GGT levels, were identified in 35.2% of patients and were significantly correlated with disease severity (*p* = 0.001), suggesting that hepatic involvement may assume a mixed pattern in more severe clinical forms. In our study, only 1.7% of the patients had more than normal total bilirubin levels, and these were not correlated with disease severity (*p* = 0.22) or hospitalization length (*p* = 0.12).

Respiratory involvement is the main cause of morbidity and mortality in adults with measles. Primary measles pneumonia, caused by viral replication in the lungs, occurs in approximately 20–30% of adults, a significantly higher percentage than that reported in children [2,13]. Bacterial superinfection pneumonia is reported in 25–40% of hospitalized cases, with *Streptococcus pneumoniae*, *Staphylococcus aureus*, and *Haemophilus influenzae* being the most common pathogens involved [15,22]. Of the cases studied, 28% developed lung-related issues. The data shows a clear trend toward viral-type interstitial pneumonia, which represented nearly three-quarters of all pneumonia instances, followed by a much smaller incidence of lobar and bronchopneumonia. No bacteriological data were available.

Acute respiratory failure occurs in approximately 10–15% of adult patients with measles pneumonia and often requires admission to intensive care units [1,23]. The clinical burden of respiratory insufficiency affected a small fraction of our study population (3.3%), notably involving twice as many children as adults. Despite these complications, the clinical course was generally favorable; almost all affected patients were stabilized via non-invasive oxygen therapy, with only one individual requiring the escalation to mechanical ventilation.

Ophthalmological complications, such as conjunctivitis and keratoconjunctivitis, occur in approximately 30–37% of cases [28,29]. Conjunctivitis was frequent in our patients—59.4% of cases, being more common in children in our study (65.7 vs. 43.7%, *p* < 0.001). No more serious ocular complications were recorded.

Neurological complications, with a frequency of 1.7–2.4%, represent the one of the most severe manifestations of the disease [1,13,15,30]. They are more common in adults than in children and represent some of the most severe manifestations of measles. In our cohort, the number of such complications was too small to make any analysis.

Mortality associated with pediatric measles remains significant, especially among children under one year of age and those who are unvaccinated. Comparative analyses of recent epidemic waves have shown mortality rates ranging from 0.3% to 3.7% among hospitalized cases, with differences being attributed to access to medical care and early diagnosis [3,22]. Measles in adults is associated with a significantly higher mortality rate than in children, with studies reporting values between 0.5% and 5%, depending on access to medical care and the patient’s immune status [3,22]. In our study, mortality was limited to a single case (0.27%), involving an 8-month-old, unvaccinated male. The clinical progression was marked by secondary bacterial pneumonia and a protracted hospital stay. Despite aggressive management in an intensive care setting, the patient did not recover, underlining the severity of bacterial superinfection as a potential driver of lethality in otherwise healthy pediatric cases

Unvaccinated or incompletely immunized healthcare workers are a vulnerable group, with numerous cases of occupational transmission reported [31,32]. No measles in our healthcare workers was reported in our hospital in 2024.

This study has several limitations. Its retrospective, the single-center design may limit generalizability, and referral bias cannot be excluded, as more severe cases are more likely to be hospitalized. Additionally, long-term outcomes were not assessed. Despite these limitations, the study benefits from a well-defined cohort, systematic data collection, and robust statistical analysis, providing valuable insights into factors associated with measles severity during the current epidemic.

## 5. Conclusions

This study provides important clinical and epidemiological insights into hospitalized measles cases during the ongoing epidemic in Romania, highlighting distinctive features of the current outbreak. The majority of cases occurred in unvaccinated or incompletely vaccinated individuals. Infants and young children represented the most affected group, emphasizing the vulnerability of populations lacking direct or indirect protection.

A key novel finding is the high frequency and severity of hepatic involvement, particularly in adults, where elevated transaminase levels were significantly more common and more pronounced than in children. This supports emerging evidence that hepatic dysfunction represents a frequent and potentially underrecognized manifestation of measles in recent epidemic waves. Adults also demonstrated stronger inflammatory responses and more frequent hematological abnormalities, suggesting a distinct clinical profile compared with pediatric cases.

These findings indicate a shift toward increased systemic involvement in contemporary measles outbreaks and underscore the urgent need to improve vaccination coverage, strengthen surveillance, and promote early recognition of severe and atypical manifestations to reduce measles-related morbidity and prevent future epidemics.

## Figures and Tables

**Table 1:** Frequency (%) of clinical manifestations in all the patients/children/adults and differences between children and adults (Fisher’s exact test).

	Total	Children	Adults	*p*-Value
Fever	93.3	93.4	93.2	0.950
Maximum temperature (average)	21.3	38.4	38.8	0.01
Cough	79.4	80.5	76.7	0.414
Rhinorrhea	51.7	59.5	32.0	<0.001
Koplick sign	55.3	56.8	51.5	0.356
Chills	36.9	31.5	50.5	0.001
Conjunctivitis	59.4	65.7	43.7	<0.001
Vomiting	17.2	14.0	25.2	0.011
Diarrhea	18.9	21.4	12.6	0.054
Abdominal pain	15.3	15.2	15.5	0.932
Adenopathy	56.9	62.2	43.7	0.001

**Table 2:** Laboratory parameters at admission in the overall cohort, children, and adults, and comparison between groups *.

Parameter	Total (*n* = 360)	Children (*n* = 257)	Adults (*n* = 103)	*p*-Value
Leukocytes (/mm^3^)	6010 ± 3500	6451 ± 3898	4896 ± 1815	<0.001
Neutrophils (%)	59 ± 19	53 ± 14	74 ± 13	<0.001
Platelets (/mm^3^)	221,409 ± 82,025	237,690 ± 85,347	180,616 ± 55,294	<0.001
CRP (mg/L)	22 (IQR 29)	16 (IQR 23)	37 (IQR 36)	<0.001
ALT (U/L)	74 (IQR 120)	44 (IQR 55)	155 (IQR 168)	<0.001
AST (U/L)	71 (IQR 84)	52 (IQR 43)	124 (IQR 121)	<0.001
GGT (U/L)	203 (IQR 150)	93 (IQR 148)	246 (IQR 241)	0.2676

Abbreviations: CRP, C-reactive protein; ALT, alanine aminotransferase; AST, aspartate aminotransferase; GGT, gamma-glutamyl transferase; IQR, interquartile range. * Continuous variables are presented as mean ± standard deviation (SD) for normally distributed data or median (interquartile range, IQR) for non-normally distributed data. Comparisons between groups were performed using the independent samples *t*-test or Mann–Whitney U test.

**Table 3:** Number of patients with ALT elevation, according to the age group.

Age Group	ALT Elevation			
	Mild	Moderate	Severe	Total
Children	84 (32.7%)	2 (0.8%)	3 (1.2%)	89 (34.6%)
Adults	39 (37.9%)	16 (15.5%)	10 (9.7%)	65 (63.1%)

## Data Availability

The original contributions presented in this study are included in the article. Further inquiries can be directed to the corresponding author.

## Abbreviations

The following abbreviations are used in this manuscript:

WHO	World Health Organization
ECDC	European Centre for Disease Prevention and Control
AST	Aspartate aminotransferase
ALT	Alanine aminotransferase
CRP	C-reactive protein
GGT	Gamma-glutamyl transferase
EU/EEA	European Union and European Economic Area
